# Study Design and Data Analysis of Artificial Pancreas Device Systems with Closed-Loop Glucose-Sensing Insulin Delivery

**DOI:** 10.1155/2021/8812695

**Published:** 2021-02-18

**Authors:** Sravya B. Shankara, Yujia Liu, Qingfeng Zheng, Jing Guo, Guixia Wang, Bo Zhang

**Affiliations:** ^1^Department of Medicine, School of Medicine, University of Massachusetts Medical School, Worcester, MA, USA; ^2^Department of Endocrinology and Metabolism, The First Hospital of Jilin University, Changchun, Jilin, China; ^3^Department of Thoracic Surgery, National Cancer Center/National Clinical Research Center for Cancer/Cancer Hospital, Chinese Academy of Medical Sciences and Peking Union Medical College, Beijing, China; ^4^Department of Health Policy and Management, School of Public Health, Peking University, Beijing, China; ^5^Department of Neurology and ICCTR Biostatistics and Research Design Center, Boston Children's Hospital and Harvard Medical School, Boston, MA, USA

## Abstract

**Objective:**

The objective of this article is to provide a high-profile review and discussion on the study design and statistical analysis of pivotal clinical trials conducted to demonstrate the safety and effectiveness of closed-loop investigational artificial pancreas device systems (APDSs) in premarket approval applications.

**Methods:**

The United States Food and Drug Administration (FDA) guidance on the content of investigational device exemption and premarket approval applications for APDSs is reviewed with special emphasis on study design and statistical analysis of the pivotal clinical trials. The two pivotal studies for the MiniMed 670G hybrid closed-loop system by Medtronic in their premarket approval application are summarized and discussed.

**Results:**

The United States FDA established detailed recommendations on the study design and statistical analysis of pivotal clinical trials for the industry that seek market investigational APDSs and for FDA scientific reviewers that regulate the device applications. The recommendations cover specifics regarding patient population, clinical endpoints, and strategies for data analysis. However, the two pivotal studies that demonstrated the effectiveness of the FDA-approved MiniMed 670G hybrid closed-loop system were not typical randomized controlled trials as per FDA recommendations.

**Conclusion:**

The development and regulation of investigational APDSs require careful and sophisticated clinical study designs and data analysis in premarket approval applications. The regulatory evaluation process of the APDSs is rather complicated since the devices consist of multiple components that collaboratively function to mimic human pancreases.

## 1. Introduction

Diabetes mellitus is a chronic disease of the body's regulation of blood glucose levels. Proper regulation of blood glucose levels is essential as glucose is an important energy source in the body, especially for the brain. Therefore, excess blood glucose levels can lead to serious health problems [1, 2]. The cause of the disease varies by type. Type I diabetes is when the pancreas produces little or no insulin, the hormone that allows for glucose to enter cells from the bloodstream, leading to hyperglycemia [3]. Type I diabetes typically manifests during childhood or adolescence, and it can develop in adults. This form of the disease can have different etiologies [4]. Type II diabetes mellitus occurs due to issues in the body's metabolism of glucose. It can either be due to the body's resistance to insulin or an inability to produce enough insulin to maintain normal blood glucose levels [3]. This form of the disease often develops slowly and is known as adult-onset diabetes, but it has become more common in children over time. There are no cures for either disease type, which is why the proper regulation of blood glucose levels is imperative for patients [3]. The artificial pancreas device system (APDS) is a device that can closely mimic a normally functioning pancreas by administering insulin at a certain threshold that can be maintained by sensing blood glucose levels [5]. This device has a significant treatment capacity for those with type I diabetes, as the artificial pancreas can compensate for the loss of pancreatic function found in most affected patients.

Currently, the United States Food and Drug Administration (FDA) guidance regarding APDS device approval [6] focuses on the use of continuous glucose monitor data for the evaluation of safety and effectiveness of the device while allowing for flexibility regarding study endpoints, indication, study size, and duration in clinical trials. The guidance, issued on November 9, 2012, details a clinical study progression that can be used by the sponsor for APDS evaluation (the term “sponsor” is used throughout this article to refer to either the manufacturers planning to market the investigational device or the investigators planning clinical research studies on the device). The guidance elaborates recommendations for low-glucose suspend systems and closed-loop control systems. Low-glucose suspend system, otherwise known as threshold suspend, is a feature that allows for an artificial pancreas device to automatically halt insulin delivery when a low blood sugar threshold is reached and to achieve full management of glucose levels. The closed-loop control system refers to an automatic control system where the delivery of insulin through the device is managed and regulated by feedback from an algorithm based on CGM data [7]. The MiniMed 670G hybrid closed-loop system by Medtronic [8] was the first second-generation, closed-loop APDS approved by the FDA to automatically monitor glucose and administer appropriate basal insulin doses in people 14 years or older with type I diabetes.

The development and regulation of an investigational APDS device require careful and sophisticated clinical study design and data analysis to demonstrate the device's safety and effectiveness. Because the APDS consists of multiple components that collaboratively function to mimic human pancreases, its evaluation process is complex. This article reviews and discusses the FDA's recommendations on the clinical study design and statistical data analysis in the investigational device exemption (IDE) and premarket approval (PMA) applications for the APDS. We also examine the pivotal clinical studies conducted and reported in the PMA Summary of Safety and Effectiveness Data (SSED) for the approval of the MiniMed 670G hybrid closed-loop system [8] and consider the concordance between the study design and statistical evidence used to the recommendations in the FDA guidance.

## 2. Artificial Pancreas Device Systems

There are three main devices used by patients with diabetes mellitus, which can be components of an APDS: (1) blood glucose devices (BGDs) that determine blood glucose levels from a finger-prick blood sample; (2) infusion pumps for continuous subcutaneous insulin infusion (CSII); (3) continuous glucose monitoring (CGM) system, which has a sensor that measures the concentration of glucose in the interstitial fluid at consistent intervals. Furthermore, it is essential to note that CGM systems are not fully adequate substitutions for BGDs, but they allow for patients to track the course of the glucose levels. However, while these devices are greatly beneficial, it can still be a struggle for patients to maintain blood glucose levels at optimal levels to prevent the risk of hypoglycemia.

**Figure 1:**
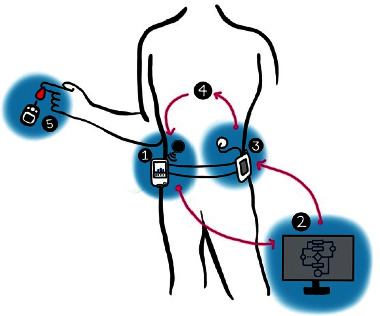
Artificial pancreas device system with four parts: (1) continuous glucose monitor (CGM); (2) APDS computer-controlled algorithm; (3) infusion pump; (4) the patient; (5) Blood glucose devices (BGDs) are used to calibrate the CGM (this figure is modified from [7]).

The primary function of an APDS is to maintain blood glucose concentrations at or near a specified range through communication between a CGM and infusion pump to automatically reduce or increase insulin infusion accordingly. There are four aspects of APDS: (1) continuous glucose monitor (CGM); (2) APDS computer-controlled algorithm; (3) infusion pump; (4) the patient (see Figure 1). The CGM's sensor is placed subcutaneously and measures the glucose levels in the interstitial fluid. The CGM displays this data and the direction and rate of change of the estimated glucose levels. The CGM's transmitter then sends all of this information to a controller. It is also crucial that the CGM is periodically calibrated with a BGD to ensure that it is detecting the most accurate glucose levels. The APDS control algorithm then analyzes the data from the CGM in an external processor, the controller. The algorithm calculates the dosing needed based on the current levels, and this information is sent to the infusion pump. The infusion pump then delivers insulin to the subcutaneous tissue and adjusts the dosage according to the received information. In all of this, the patient plays a central role in the functioning of the APDS. Blood glucose levels are ever-changing and fluctuate according to the patient's diet, activity level, and metabolism capacity.

There are three main categories of APDS that are currently used: Threshold Suspend Delivery System, Control-to-Range (CTR) system, and Control-to-Target (CTT) system [6]. The Threshold Suspend Device System adjusts insulin dosing only if the sensor value reaches a predetermined lower threshold of measured interstitial glucose. However, this device does not take any action if the sensor value is above the determined threshold. On the other hand, the CTR system adjusts insulin levels to maintain a predetermined higher and lower threshold. At the same time, it will not take any action when the glucose levels are within the thresholds. With the use of either of these two devices, patients should still monitor their blood glucose concentration, set appropriate basal rates for the insulin pump, and take premeal bolus insulin to maintain control of their glucose levels. The CTT system is unique because it requires no further interaction of the user beyond the CGM's calibration. The CTT system is fully automated to maintain set target glucose levels at all times.

There are two CTR and CTT system subtypes depending on what drug(s) is being delivered and how the drug affects glucose concentrations: insulin-only system and bihormonal control system [6, 7]. While an insulin-only system achieves a target glucose level by increasing or decreasing insulin dosage, a bihormonal control system maintains thresholds through the use of an APDS control algorithm(s) for the infusion pump. This allows for the administration of insulin to lower glucose levels and another hormone, such as glucagon, to increase blood glucose levels. Therefore, the bihormonal control system mimics the regulation of blood glucose levels by the pancreas more precisely than an insulin-only system.

The FDA guidance indicates that “the APDS is intended for patients with type 1 diabetes for the subcutaneous infusion of insulin and the continuous measurement of interstitial glucose to aid in the management of their disease. The APDS automatically adjusts insulin delivery in response to CGM values that have exceeded or are predicted to exceed the bounds of a prespecified blood glucose range. The APDS is intended to assist the patients in managing their disease” [8].

## 3. Study Design and Data Analysis of Clinical Studies for Investigating the Artificial Pancreas Device Systems

### 3.1. Study Design of Pivotal Clinical Studies

The pivotal clinical studies for an investigational APDS function to collect data to support the device's safety and effectiveness. These studies should be performed with the final APDS device for its actual use in a real-world environment. The FDA recommends that sponsors compare clinical outcomes between patients using a sensor-augmented pump control device and patients using the APDS. It is suggested that the control group uses currently available technology for there to be a proper analysis of the new APDS' technology and algorithm. FDA advises that a robust pivotal clinical trial design to validate an investigational APDS should be either a randomized cross-over design or a randomized parallel design in an outpatient setting. The standard of care should be given to the patients in the control group by performing finger stick blood glucose tests to determine proper therapy. According to these results, the patients in the test group should be treated by a systematic adjustment as directed by the instructions of the APDS.

The patient population in the pivotal clinical studies should be selected according to the intended use of the APDS device because the chosen study population can largely influence the study design, sample size, duration of follow-up, and final approved device indications. The FDA recommends that sponsors consider these factors while enrolling patients for the initial subject population: subjects experienced with insulin pumps for more than 6 months, subjects willing to perform more than 4 finger stick blood glucose measurements daily, subjects willing to perform manufacturer-required sensor calibrations, subjects willing to wear the system more than 6 days per week, subjects willing to keep a minimum log of sick days, days with exercise, or symptoms of low and high blood glucose episodes and medications. Suppose sponsors aim to improve study efficiency by reducing study sample size and study duration; the study patients may be enriched by targeting subjects with a high percentage HbA1c and frequent hypoglycemia despite aggressive attempts at improved glycemic control, subjects with purposeful maintenance of high HbA1c to avoid any hypoglycemia, and subjects with frequent hypoglycemia. Furthermore, the FDA recommends that subjects be older than 18 years of age to ensure that patients can respond to device problems. However, the FDA is also keen on promoting the development of a safe and effective APDS for subjects younger than 8 years of age.

Clinical study endpoints to evaluate the APDS performance will mainly target CGM data, the measurement, or estimation of blood glucose levels. Therefore, the FDA determines that it is acceptable and appropriate for CGM data to be used to evaluate an investigational APDS. While endpoints, indication for use, and intended marketing claims will differ for each particular device, the endpoints should numerically reflect the degree of the safety and effectiveness of the APDS. The FDA recommends measurements of hypoglycemia and hyperglycemia, level of glycated hemoglobin (HbA1c), or Time in Range (TIR) for potential endpoints of these studies. In regard to hypoglycemia and hyperglycemia, potential endpoints include “number of hypoglycemic and hyperglycemic events or event rate; time spent in hypoglycemia or low glycemic concentrations and hyperglycemic or high glycemic concentration events; average duration for all hypoglycemic or low glycemic concentration events and hyperglycemic or high glycemic concentration events; or mean area under the curve (AUC) for all hypoglycemic events less than 70 mg/dL and hyperglycemic events greater than 240 mg/dL” [6]. Another potential primary effectiveness endpoint is the level of HbA1c, as it estimates the average glycemic exposure of red blood cells over 90 days. The endpoint TIR that determines how successful an APDS can keep glucose within a predefined range can also be adopted as the primary effectiveness endpoint in the pivotal clinical studies for the APDSs. While assessing TIR, it is essential to also examine the effect of the device on clinical symptoms, glucose values above and below desired ranges, and examine the TIR in relation to other glycemic control markers.

The safety endpoints for an investigational APDS should be evaluated to ensure that the device does not increase the incidence of severe hypoglycemia or severe hyperglycemia or diabetic ketoacidosis. Therefore, some safety endpoints recommended by the FDA include “severe hyperglycemia and elevated ketones; diabetic ketoacidosis; number of CGM-defined hyperglycemic events; mean AUC above 240 mg/dL as calculated from CGM readings; HbA1c above a predefined accepted increase that may occur as a result of reduction of hypoglycemia or from inappropriate pump suspensions or decreased insulin delivery; percentage of CGM readings in the higher hyperglycemic ranges; severe hypoglycemia; number of CGM-defined hypoglycemic events; mean AUC below 60 or 70 mg/dL as calculated from CGM readings; or percentage of CGM readings in the hypoglycemic range less than 60 or 70 mg/dL” [6]. Other potential endpoints, again based on the specific device and its intended use, can include “incidence of catheter blockage within each group; capillary blood glucose values above and below the defined hypoglycemia and hyperglycemia thresholds; fasting whole blood ketone concentrations within each group, evaluating elevated beta-hydroxybutyrate concentrations; glycemic variability; incidence, severity, and timing of CGM-events for hypoglycemia and hyperglycemia; safety and effectiveness subgroup analysis, such as for pediatric subjects; quality of life; weighted mean AUC of CGM-events for hypoglycemia and hyperglycemia” [6].

### 3.2. Data Analysis of Pivotal Clinical Studies

The FDA recommends certain general considerations for data analysis, including patient demographics, medical history, and other important baseline characteristics, summarized using descriptive statistics and frequency tables as appropriate. It is suggested that patient accountability and withdrawals from the study's treatment phase should also be reported. Using appropriate models, typically a linear model for a continuous variable and a logistic regression for a binomial variable, the effects of carryover, sequence site, baseline variables, and prognostic variables should be tested.

The data analysis of primary effectiveness endpoints in the pivotal clinical studies for the APDS devices should be primarily between-group comparison with respect to the primary endpoints. The effects of the covariates must be adjusted through appropriate statistical models, as long as these covariates are found to confound the primary endpoints. The data analysis of the secondary effectiveness endpoints is recommended to be conducted through descriptive statistics. Data analysis can be conducted through inferential statistics when the sponsor intends to make labeling claims for any of the endpoints. However, the sponsor must clearly specify the hypothesis, statistical analysis, and success criteria on these secondary endpoints in the study protocol. Multiplicity adjustment procedures for controlling the type 1 error rate should be used (e.g., Bonferroni correction for controlling the familywise error rate or Benjamini-Hochberg procedure for controlling the false discovery rate).

In terms of performing hypothesis testing on endpoints' effectiveness, it is typical that two populations are used: Intention to Treat (ITT) population and the Per Protocol (PP) population. For the analysis of primary endpoints, the ITT population is widely preferred. The ITT population should be composed of all randomized subjects, while the PP population consists of all randomized subjects who complete the treatment period without major protocol deviations. Therefore, the sponsor must define the major protocol deviations in the PP population with details in the study protocol. It is also recommended that, in the study protocol, the sponsor clearly states the hypothesis for each primary endpoint and defines the overall success criteria of the study. The statistical plan for the pivotal clinical trial should be defined in advance in the study protocol as well. If the study is designed to assess either noninferiority or superiority with a margin between the APDS and control groups, the sponsor should propose and justify their noninferiority or superiority margin prior to the beginning of their pivotal clinical studies.

Although the missing data are usually inevitable in the pivotal clinical trials, patient withdrawals and loss to follow-ups should be minimized as much as possible while designing the pivotal clinical trials and throughout the trials. Missingness should be thoroughly documented with the reason for missing and the specific treatment group. The FDA recommended that, if there are missing data in primary endpoints, they should be appropriately imputed in the ITT population analysis. A sensitivity analysis, using not only one but various methods, should be planned in the protocol and conducted afterward to evaluate the impact of missing data. The sensitivity analysis may include PP analysis and be based upon last observation carried forward, multiple imputations, all missing as failures or success, worst-case scenario, best case scenario, tipping point, and others [9].

For the data analysis of safety endpoints, descriptive statistics of all adverse events should be presented for both investigational studies and premarket submissions. The FDA recommends that the descriptive statistics of these subgroups should also be summarized. Furthermore, the safety population for the pivotal study of an APDS must include all randomized subjects.

## 4. Design and Analysis of Clinical Trials for the MiniMed 670G System

The MiniMed 670G system, manufactured by Medtronic (Medtronic Public Limited Company), is an artificial pancreas device system approved by the FDA on September 28, 2016. This system comprises five devices: MiniMed 670G insulin pump, the Guardian Link (3) Transmitter, the Guardian Sensor (3), One-Press Serter, and the Contour NEXT Link 2.4 Glucose Meter. The MiniMed 670G system is “intended for continuous delivery of basal insulin at user-selectable rates and administration of insulin boluses in user-selectable amounts for the management of type 1 diabetes in persons, fourteen years of age and older, requiring insulin as well as for the continuous monitoring and trending of glucose levels in the fluid under the skin.” The MiniMed 670G system adopts “SmartGuard technology.” Medtronic's MiniMed 670G is considered to be the very first FDA-approved closed-loop, fully automated artificial pancreas device that allows computer programs to automatically adjust delivery of basal insulin according to CGM sensor glucose values and to “suspend delivery of insulin when the sensor glucose value falls below or is predicted to fall below predefined threshold values” [8].

Medtronic designed and performed two pivotal clinical studies in the United States to demonstrate the safety and effectiveness of the device for diabetes patients. One pivotal study, Investigational Device Exemption or IDE G140053, was a multicenter, prospective, single-sample correlational study without any control group to evaluate the performance measurement of the Guardian Sensor over 7 days. With proper inclusion and exclusion criteria, this study enrolled a total of 89 adolescents and adults with type 1 or type 2 diabetes mellitus between the ages of 14–75 years from 6 investigational sites. The subjects wore a receiver, the Guardian Link Transmitter, and the Guardian Sensor for a 7-day study period, during which each subject participated in a three-day “frequent sample testing” in Day 1, Day 3, and Day 7. During these three days, intravenous blood samples were obtained from the subjects every 5 to 15 minutes in a period of approximately 12 to 14 hours. Then, the samples were analyzed for plasma blood glucose levels using the Yellow Springs Instrument 2300 Stat Plus Glucose Lactate Analyzer, referred to as the comparator method (CM). The subjects were advised to continue with their previous diabetes regimen to manage their diabetes instead of relying on the investigational devices. The accuracy of the Guardian Sensor was assessed by comparing the Guardian Sensor values to the ones obtained from the CM.

To support the accuracy of the Guardian senor, descriptive statistics were reported on percent difference of the CGM values (using the Guardian Sensor) with respect to the CM values, percent of the CGM values that fell within 15, 20, 30, 40, and >40 mg/dL of the CM values, number and percentage of the CM values collected while the CGM values were less than 40 mg/dL or greater than 400 mg/dL, and concurrence and agreement rate of the CGM values and the CM values. Overall, these numerical results presented substantial evidence to support the Guardian Sensor's accuracy in automatically adjusting basal insulin rates. In this study, some subjects wore two Guardian Sensors. The precision of the sensor system was assessed by comparing sensor values to each other in these subjects. Data from two parallel sensors provided 30,350 pairs of CGM measurements with a mean percent absolute relative difference of 9.07% with a coefficient of variation of 6.5%.

Another pivotal clinical study (IDE G140167) was a multicenter, single-arm, home and hotel clinical study to evaluate the safety of the MiniMed 670G system and its algorithm with the Guardian Sensor. This pivotal study recruited 126 patients between the ages of 14 and 75, with type 1 diabetes at 10 investigational sites. The subjects used the 670G pump with the Guardian Link Transmitter, the Guardian Sensor, and infusion sets for around 3.5 months. The study included a 2-week run-in period, a 3-month at home use period, and a 5-day and 6-night hotel study during month 1, 2, or 3 of the study. The device was instructed to be used in the Auto Mode for the entire 3 months at home study by the subjects. This study was a descriptive study to evaluate the safety of the MiniMed 670G system with the Auto Mode. Therefore, there were no statistically powered endpoints in the pivotal study, nor was there any hypothesis testing. Although the effectiveness of the system was also assessed by descriptive statistics on HbA1c level, the total daily dose of insulin, time spent in Auto Mode versus time spent in Manual Mode, time in different glucose range, time in the hyperglycemic range, and so on without comparison to alternative treatments, the emphasis of the study is still the safety evaluation. Safety endpoints were serious adverse events, serious adverse device events, unanticipated adverse device effects, incidence of severe hypoglycemia, and incidence of diabetic ketoacidosis. The results of the study showed no reports of unanticipated adverse device effects, no reports of diabetic ketoacidosis, and no reports of severe hypoglycemia events. At the same time, there were 24 severe hyperglycemia events reported. There were four serious adverse events reported of appendicitis, bacterial arthritis of the right wrist, *C*. *difficile* diarrhea, and worsening rheumatoid arthritis. Furthermore, there were four procedure-related adverse events: thrombophlebitis, pain, irritation or bruising, and pain at the intravenous site.

## 5. Discussion and Conclusions

From a regulatory standpoint, this article reviews the FDA's recommendations on study design and data analysis of the pivotal clinical trials for evaluating the safety and effectiveness of an investigational APDS for automated regulation of blood glucose levels of patients with type 1 diabetes. While the statistical analysis is relatively standard in these clinical trials [10], there are unique features for the selection of patient population and clinical endpoints in study design [6].

The Juvenile Diabetes Research Foundation defined three generations of nonclosed-loop and closed-loop APDSs [7, 11]. An APDS is “closed-loop,” meaning that the system is fully automated that integrates a CGM and an insulin pump with a digital controller to achieve continuous management of type 1 diabetes. First-generation APDSs are nonclosed-loop, not fully automated systems with primary functions of low-glucose suspend and predictive low- and high-glucose suspend. Second-generation APDSs are automated insulin delivery systems. The early stage of the second-generation APDSs is hybrid closed-loop devices at all times with mealtime manual assist bolus, while the late stage of the second-generation APDSs is fully automated insulin delivery systems. Third-generation systems are fully automated multihormonal delivery devices. These devices administrate a secondary glucoregulatory hormone in addition to insulin. The MiniMed 670G system is the first fully automated, second-generation APDS approved by the FDA and commercially available on the United States market.

Second-generation APDSs are equipped with fully automated insulin delivery and predictive low-glucose management. Compared to the first-generation sensor-augmented APDSs, patients can benefit from second-generation APDSs due to a potential improvement in control of HbA1c values, less time and number of events with hypoglycemia and hyperglycemia, and more time within a normal range of sensor glucose. However, there are still risks associated with the second-generation APDSs' full automation and predictive suspension: improper suspension or decrease in insulin delivery due to software error or erroneous CGM data or inappropriate increase in insulin delivery or suggestions that patients take additional insulin due to a software error or erroneous CGM data. As a result, hyperglycemia, ketosis, ketoacidosis, or hypoglycemia may occur because of erroneously willing or unwilling off label use of the device or software malfunction. The risks with the predictive low-glucose management are that the predictive suspend feature may inappropriately suspend or resume insulin due to a software defect or erroneous CGM data, which inaccurately detects impending hypoglycemia or hypoglycemia. Yet, a first-of-its-kind second-generation APDS, the MiniMed 670G system, was approved as the FDA concluded its benefits outweigh its risks.

The FDA approval of second-generation APDSs gives patients and healthcare providers an opportunity for continuous glucose tracking and trending information that is not feasible using traditional blood glucose monitoring. Using the second-generation APDSs, patients and healthcare providers can now review the tracking and trending data by time of day, such as daytime or nighttime, when fewer finger sticks are conducted.

Because there were two first-generation APDSs developed by the same manufacturer [12, 13], the designed pivotal clinical trials for the MiniMed 670G system in its PMA substantially deviated from the recommendations deliberated in the APDS guidance [6]. While the sponsor closely follows the FDA's recommendations on evaluating the safety of the device, the sponsor conducted a pivotal trial to evaluate the diagnostic effectiveness of the system compared to the CM with a single-arm observational study instead of a two-group randomized controlled trial or cross-over trial. This reflects the flexibility of medical device clinical trials in the regulatory process.

The FDA guidance also presents suggestions for the development of feasibility studies for the APDS systems [6]. While not all APDS systems need to go through feasibility studies, these are designed to test specific aspects of the APDS functionality and performance and to collect preliminary data on safety. As feasibility studies aim to be exploratory for device development and to support the APDS functions without unexpected safety concerns, feasibility studies are expected to have a short duration and small patient population and to be descriptive. It is imperative that there are identifiable goals and prespecified success criteria for feasibility studies. Future research on the study design and data analysis of the clinical trials for investigational APDSs is not unique and should be in alignment with the pursuit of developing new methodologies and designs in clinical trials. As a regulatory agency, the FDA should pay attention to their regulation on the use of observational studies for investigating the effectiveness and safety of the APDSs.

## Data Availability

No original data were used to support the results.
